# Condylomes anaux de l'enfant

**DOI:** 10.11604/pamj.2014.17.1.3736

**Published:** 2014-01-06

**Authors:** Maha Mael-ainin, Karima Senouci

**Affiliations:** 1 Service de Dermatologie, CHU Ibn Sina, Université Mohamed V, Souissi, Rabat, Maroc

**Keywords:** Condylomes, verrues, région ano-génitale, enfant, warts, verruca, anogenital area, child

## Image en médicine

Les condylomes correspondent à des verrues ano-génitales secondaires à une infection par Human Papilloma Virus (HPV). On distingue 3 formes cliniques qui peuvent être associées: les condylomes acuminés, papuleux et plans. Les condylomes acuminés sont les classiques crêtes de coq, tumeurs exophytiques à surface verruqueuse et hyperkératosique de couleur érythémateuse, grisâtre, ou chaire. Les condylomes papuleux se manifestent sous formes de lésions rosées ou pigmentées, lisses ou hyperkératosiques, isolée ou en nappe. Les condylomes plans sont des macules de couleurs rosées ou blanches, parfois infracliniques révélés par leur blanchiment suite à l'application de l'acide acétique à 5%. Le traitement est non consensuel, les récidives son fréquentes. Chez l'enfant, la prise en charge des condylomes ano-génitaux doit passer d'abord par l'identification du mode de contamination. Dans la majorité des cas il s'agit d'une transmission manuportée soit par auto ou hétéro-inoculation à partir de verrues cutanées. En l'absence d'une explication évidente des condylomes ou en cas d'arguments faisant suspecter des sévices sexuels, il est indispensable de faire hospitaliser l'enfant, de rechercher d'autres infections sexuellement transmissibles et de faire une enquête psychologique et sociale. Nous rapportons le cas d'un nourrisson de sexe féminin, âgé de 20 mois, sans antécédents pathologiques. La patiente présentait des lésions papuleuses péri-anales, érythémateuses et confluentes en nappe, sans signes associés. L'examen des mains de la mère a objectivé la présence de verrues vulgaires. Le diagnostic de condylomes anaux de transmission non sexuelle a été retenu. La patiente a été mise sous vaseline salicylée à 5% à raison d'une application par jour avec une bonne évolution après 6 semaines de traitement.

**Figure 1:**
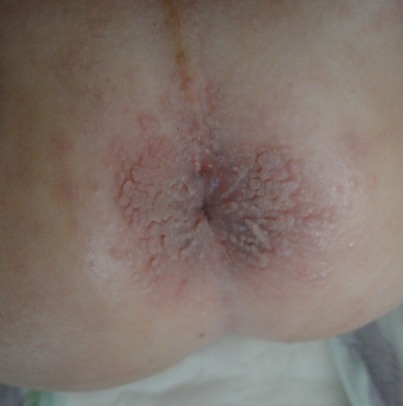
Lésions papuleuses péri-anales érythémateuses et confluentes en nappe

